# Supplementary material for: Lipids and ions traverse the membrane by the same physical pathway in the nhTMEM16 scramblase
Source: eLife. 2017 Sep 16;6:e28671. doi: 10.7554/eLife.28671 (PMC5628016; doi:10.7554/eLife.28671)
Supplement: Transparent reporting form [file elife-28671-transrepform.docx]

***eLife’s* transparent reporting form**

We encourage authors to provide detailed information *within their submission* to facilitate the interpretation and replication of experiments. If you have any questions, please contact us: [editorial@elifesciences.org](mailto:editorial@elifesciences.org).

**Sample-size estimation**

- You should state whether an appropriate sample size was computed when the study was being designed
- You should state the statistical method of sample size computation and any required assumptions
- If no explicit power analysis was used, you should describe how you decided what sample (replicate) size (number) to use

Please outline where this information can be found within the submission (e.g., page numbers or figure legends), or explain why this information doesn’t apply to your submission:

For electrophysiology and phospholipid scrambling experiments, the sample size for each measurement was chosen based on generally applied criteria for electrophysiology and imaging data. The corresponding errors, calculated as s.e.m. were small and shown in Figures 4D, 5C, and 5D. Measurements were based on 4 – 29 cells as described in the figure legends.

**Replicates**

- You should report how often each experiment was performed
- You should include a definition of biological versus technical replication
- The data obtained should be provided and sufficient information should be provided to indicate the number of independent biological and/or technical replicates
- If you encountered any outliers, you should describe how these were handled
- Criteria for exclusion/inclusion of data should be clearly stated
- High-throughput sequence data should be uploaded before submission, with a private link for reviewers provided (these are available from both GEO and ArrayExpress)

Please outline where this information can be found within the submission (e.g., page numbers or figure legends), or explain why this information doesn’t apply to your submission:

Each experiment was repeated on at least 2 separate independent transfections and at least 2 control and 2 mutant cells were measured from each transfection. Results from different experiments were pooled and averaged. All cells were included in analysis except cells that were deleted for technical reasons such as excessive leak current or cells where the seal was lost before completion of the experiment. No outliers were eliminated.

Data handing is described in the Methods (pages 33-35) and Figure legends (pages 48-51).

The sample sizes of electrophysiology data and scrambling data is shown in the Figures 4, 5, and 6 and in the associated figure legends (pages 48-51). Data represents averages of independent recordings from at least 4 independent cells.

**Statistical reporting**

- Statistical analysis methods should be described and justified
- Raw data should be presented in figures whenever informative to do so (typically when N per group is less than 10)
- For each experiment, you should identify the statistical tests used, exact values of N, definitions of center, methods of multiple test correction, and dispersion and precision measures (e.g., mean, median, SD, SEM, confidence intervals; and, for the major substantive results, a measure of effect size (e.g., Pearson's r, Cohen's d)
- Report exact p-values wherever possible alongside the summary statistics and 95% confidence intervals. These should be reported for all key questions and not only when the p-value is less than 0.05.

Electrophysiology data is reported as means plus/minus SEM from at least 4 independent cells. Current voltage-relationships are shown for typical cells of at least 4 independent recordings. Imaging of phospholipid scrambling in Fig. 4D and 6C is reported as the percentage of cells studied overall. Each mutation was studied in 4 - 29 independent cells.

Please outline where this information can be found within the submission (e.g., page numbers or figure legends), or explain why this information doesn’t apply to your submission:

(For large datasets, or papers with a very large number of statistical tests, you may upload a single table file with tests, Ns, etc., with reference to page numbers in the manuscript.)

**Additional data files (“source data”)**

- We encourage you to upload relevant additional data files, such as numerical data that are represented as a graph in a figure, or as a summary table
- Where provided, these should be in the most useful format, and they can be uploaded as “Source data” files linked to a main figure or table
- Include model definition files including the full list of parameters used
- Include code used for data analysis (e.g., R, MatLab)
- Avoid stating that data files are “available upon request”

Please indicate the figures or tables for which source data files have been provided:

N/A
